# Efficient Fabrication of Disordered Graphene with Improved Ion Accessibility, Ion Conductivity, and Density for High‐Performance Compact Capacitive Energy Storage

**DOI:** 10.1002/advs.202405155

**Published:** 2024-08-09

**Authors:** Gangqiang Liu, Xiangming Li, Congming Li, Qinwen Zheng, Yingche Wang, Ronglin Xiao, Fei Huang, Hongmiao Tian, Chunhui Wang, Xiaoliang Chen, Jinyou Shao

**Affiliations:** ^1^ Micro‐/Nano‐Technology Research Center State Key Laboratory for Manufacturing Systems Engineering Xi'an Jiaotong University Xi'an Shaanxi 710049 China; ^2^ Xi'an Institute of Electromechanical Information Technology Xi'an Shaanxi 710065 China; ^3^ Shaanxi Coal Chemical Industry Technology Research Institute Co., Ltd Xi'an Shaanxi 710075 China; ^4^ Frontier Institute of Science and Technology (FIST) Xi'an Jiaotong University Xi'an Shaanxi 710049 China

**Keywords:** compact supercapacitor, disordered graphene, high density, porous structure

## Abstract

High‐performance compact capacitive energy storage is vital for many modern application fields, including grid power buffers, electric vehicles, and portable electronics. However, achieving exceptional volumetric performance in supercapacitors is still challenging and requires effective fabrication of electrode films with high ion‐accessible surface area and fast ion diffusion capability while simultaneously maintaining high density. Herein, a facile, efficient, and scalable method is developed for the fabrication of dense, porous, and disordered graphene through spark‐induced disorderly opening of graphene stacks combined with mechanical compression. The obtained disordered graphene achieves a high density of 1.18 g cm^−3^, sixfold enhanced ion conductivity compared to common laminar graphene, and an ultrahigh volumetric capacitance of 297 F cm^−3^ in ionic liquid electrolyte. The fabricated stack cells deliver a volumetric energy density of 94.2 Wh L^−1^ and a power density of 13.7 kW L^−1^, representing a critical breakthrough in capacitive energy storage. Moreover, the proposed disordered graphene electrodes are assembled into ionogel‐based all‐solid‐state pouch cells with high mechanical stability and multiple optional outputs, demonstrating great potential for flexible energy storage in practical applications.

## Graphical abstract

Spark‐induced fast expansion and reduction allows for the formation of porous graphene, which has disorderly connected graphene sheets, enabling high ion conductivity and high ion accessibility while achieving high density by mechanical compression. The disordered graphene‐based supercapacitor delivers an ultrahigh energy density of 94.2 Wh L^‐1^ and a power density of 13.7 kW L^‐1^, representing a milestone in capacitive energy storage.

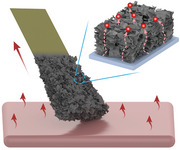

## 1 Introduction

**Figure 1:**
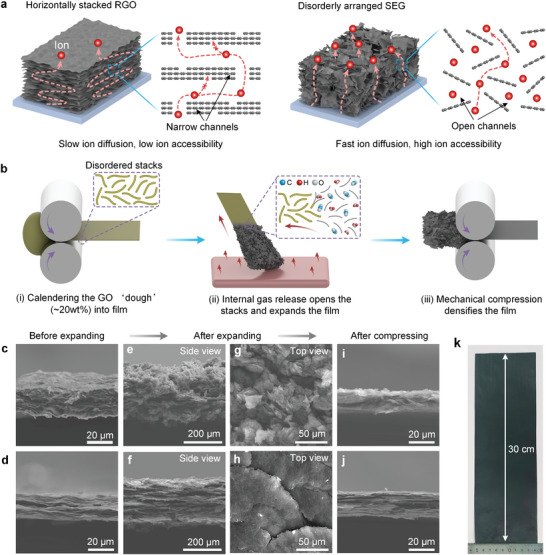
Design and fabrication of disordered SEG electrode film. a) Illustration of the ion transport and accessibility in common horizontally stacked RGO films (left) and disordered arranged SEG films (right). b) Schematic illustration of the fabrication process of disordered SEG films. i) Calendering the GO “dough” into a disordered GO film. ii) Transformation of the GO film into a porous and disordered graphene “foam” through contact with a hot plate to trigger a spark‐induced expansion and reduction reaction. iii) Compacting the porous graphene by controllable mechanical compression. c–j) SEM images of the different graphene electrode films. Disordered graphene films before expansion (c, side view), after expansion (e, side view, and g, top view), and after compression (i, side view). Laminar graphene films before expansion (d, side view), after expansion (f, side view, and h, top view), and after compression (j, side view). Of note, the presented films (c and d) have already been reduced by annealing. k) A photograph showing a prepared centimeter‐scale free‐standing electrode film.

The scalable and sustainable manufacturing of dense yet porous electrode films with high ion‐accessible surface area and fast ion diffusion capability is crucial for large‐scale capacitive energy storage with high volumetric energy and power densities requiring rapid response and limited‐space, such as grid power buffers, electric vehicles, and portable electronic devices.^[^ ^1^, ^2^, ^3^, ^4^ ^]^ Graphene is a widely used electrode material for high‐performance capacitive energy storage owing to its large surface‐area‐to‐volume ratio, high intrinsic electrical conductivity, and excellent chemical stability.^[^ ^5^, ^6^, ^7^ ^]^ However, traditional methods used to fabricate graphene electrodes, such as vacuum filtration and spin coating often result in horizontal restacking of 2D graphene sheets induced by capillary force and strong π‐π interaction,^[^ ^8^, ^9^, ^10^ ^]^ resulting in graphene electrodes with tortuous ion diffusion pathways and narrow interlayer spacing (**Figure** **1**a, left). In turn, features like tortuous ion diffusion pathways impede ion transport kinetics, thereby limiting the high‐rate performance of the graphene‐based electrodes. Moreover, the ion diffusion pathways across the electrodes grow exponentially with the increase in electrode thickness, significantly compromising the rate capability of thicker electrode films.^[^ ^11^, ^12^ ^]^ On the other hand, the narrow interlayer spacing not only impedes ion diffusion but also restricts ion accessibility within the electrodes, limiting the specific capacitance and energy density of capacitive energy storage devices.

To enhance the ion transport kinetics and improve ion accessibility of graphene electrodes, tremendous research efforts have been devoted but often come at the expense of sacrificing electrode density for low volumetric performance. For instance, strategies like laser scribing,^[^ ^13^, ^14^ ^]^ chemical etching,^[^ ^15^, ^16^ ^]^ or graphene gelation^[^ ^17^, ^18^ ^]^ could increase film porosity for fast ion diffusion and high ion accessibility due to the created large pores serving as transport highways and electric double‐layer (EDL) sites for ions. However, the resulting porous graphene electrode films generally exhibit low density, typically varying from less than 0.1 g cm^−3^ to no more than 0.6 g cm^−3^. This limits the volumetric capacitance to usually no more than 100 F cm^−3^ and induces low device volumetric energy density generally no more than 50 Wh L^−3^. Vertically aligning 2D sheets by mechanical shearing,^[^ ^19^, ^20^ ^]^ directionally freezing,^[^ ^21^, ^22^ ^]^ or plasma‐enhanced chemical vapor deposition (PECVD)^[^ ^23^, ^24^ ^]^ may enable directional ion transport for significantly reduced tortuosity of ion diffusion pathways. However, existing vertically aligned graphene fabrication methods often suffer from complicated manufacturing processes or low fabrication efficiency, posing challenges for scalable production. In addition, vertically aligned graphene also suffers from low density due to the generation of large pores during the vertical alignment or growth process.

To construct graphene films with both high porosity and high density, the top‐down method involving an initial creation of an open porous structure followed by shrinkage to reduce large pores and improve density, has been considered an effective strategy. For instance, the use of mechanical compression can densify H_2_O_2_‐etched porous graphene frameworks to result in improved density from ≈ 0.012 to 0.71 g cm^−3^, enabling significantly improved ion transport and ion accessibility in a compact form.^[^ ^25^ ^]^ In another study, dense yet porous liquid‐mediated graphene hydrogel films can be fabricated using volatile/no‐volatile liquids as intercalants to prevent the restacking of graphene sheets and construct porous internal pathways, combined with capillary compression to achieve a high packing density of 1.33 g cm^−3^.^[^ ^26^ ^]^ In addition, a novel strategy involving finely tuning pore size by introducing exfoliated graphene as intercalants during the film self‐assembly process has also been proposed to optimize the trade‐off between density and porosity, reporting a high volumetric capacitance of 203 F cm^−3^ and moderate electrode density of 0.94 g cm^−3^.^[^ ^27^ ^]^ However, introducing intercalations through no‐volatile liquid or exfoliated graphene highly depends on the availability of monolayer graphene (oxide) raw material, currently difficult to produce on a large scale at an industry‐acceptable cost.^[^ ^28^ ^]^ Moreover, the long‐range orientation of 2D graphene sheets parallel to current collectors barely changes, resulting in high electrode tortuosity and limited rate performance.

Herein, a novel facile, efficient, and scalable method is developed for the fabrication of disordered graphene through an expansion‐compression strategy, enabling a dense integration of graphene with high ion accessibility and fast ion diffusion capability. By using spark‐induced expansion of graphene stacks, a porous and disordered graphene can be efficiently fabricated. This porous, and disordered spark‐expanded graphene (SEG) can achieve a high density of 1.18 g cm^−3^ through mechanical compression while retaining its microscopic porous characteristics, making it an ideal electrode material for high‐performance compact capacitive energy storage applications. The obtained dense, disordered SEG electrodes exhibit sixfold enhanced ion conductivity over common laminar structured electrodes with an ultrahigh volumetric capacitance of 297 F cm^−3^ in ionic liquid electrolyte. The stack cells deliver a volumetric energy density of 94.2 Wh L^−1^ and a power density of 13.7 kW L^−1^, representing a milestone in capacitive energy storage. Moreover, all‐solid‐state flexible pouch cells fabricated by assembling the disordered SEG films using ionogel electrolyte demonstrate high mechanical flexibility and cycling stability, highlighting great potential for flexible energy storage applications.

## 2 Results and Discussion

### 2.1 Design and Preparation of Disordered SEG

A schematic illustration of disordered SEG as an ideal electrode material for high‐performance capacitive energy storage is shown in Figure 1a. Common laminar reduced graphene oxide (RGO) electrode film exhibits tortuous ion diffusion pathways and narrow interlayer spacing due to the horizontal restacking behavior of 2D material during the self‐assembly process, resulting in sluggish ion diffusion and low ion accessibility (Figure 1a, left). By comparison, the disordered SEG electrode film exhibits shortened ion diffusion pathways and improved ion accessibility induced by the uniquely formed architecture consisting of disorderly arranged graphene sheets in an interconnected 3D porous graphene network fashion (Figure 1a, right).

To realize this architecture, graphene oxide (GO) dough films are first prepared via a calendering process (Figure 1b–i). Briefly, GO slurry (≈4 wt.%) is first stirred to facilitate the evaporation of water until a dough‐like state (≈20 wt.%, Figure S1, Supporting Information) is obtained with GO sheets disorderly agglomerated (Figure S2, Supporting Information). Such formed GO “dough” possesses good cohesiveness and viscoelastic properties, allowing its shaping into various forms without the need for binders (Figure S3, Supporting Information), consistent with the literature.^[^ ^29^ ^]^ Taking advantage of this merit of GO “dough”, it can be readily calendered into a thickness‐controllable GO film, as shown in Figure S4 (Supporting Information). Importantly, the close agglomeration of GO sheets also significantly enhances van der Waals force and viscous drag force interactions, providing high elastic energy to resist ordered alignment during the calendering process. The obtained GO dough film exhibits a comparatively disordered internal arrangement, distinct from the typical laminar film structure, as evidenced by scanning electron microscopy (SEM) images (Figure 1c,d). Obviously, the laminar film in Figure 1d appears flat with horizontally stacked sheets, while the GO sheets in Figure 1c look more curved (or crimped) and tangled together in a disordered manner to form the calendered GO dough film. The obviously weaker diffraction peak at ≈12.7° of the calendered GO dough film in the X‐ray diffraction (XRD) pattern also indicates a more disordered sheet orientation compared to that of laminar GO film (Figure S5, Supporting Information).

After spark‐induced expansion and reduction reaction, the as‐prepared GO dough film is further transformed into a porous graphene “foam” with 3D interconnected disordered graphene sheets (Figure 1b–ii). Upon contact of the GO dough film with a hot plate at 500 °C, a spark immediately appears and quickly spreads across the whole film, accompanied by a change in film color from dark brown to matte dark black (Figure S6; Movie S1, Supporting Information). The rapid decomposition and removal of oxygen‐containing functional groups present in the GO sheets under instantaneous superheating generates a large amount of gas, including CO_2_ and H_2_O vapor.^[^ ^30^ ^]^ The quick release of generated gases creates abundant open pores within the film, expanding it to the porous graphene “foam”. X‐ray photoelectron spectroscopy (XPS) measurement reveals a significantly increased C/O atomic ratio for sparked graphene “foam” (6.56) compared with GO (2.22), suggesting a significant reduction in O after the spark reaction. In the deconvoluted C 1s spectra, the intensity corresponding to the C─C peak (284.8 eV) increases by 57.8% after the spark reaction (Figure S7, Supporting Information). Additionally, in Fourier transform infrared (FT‐IR) spectroscopy, characteristic peaks of oxygen‐containing groups, such as hydroxyl (─OH, broad peak beyond 3000 and 1390 cm^−1^), carbonyl (C═O, 1730 cm^−1^), and epoxy (C─O─C, 1039 cm^−1^), become quite weak or even undetectable after the spark reaction, confirming that most of the oxygen‐containing groups have been removed (Figure S8, Supporting Information). The resulting porous SEG consists of numerous interconnected graphene agglomerations with graphene sheets disorderly arranged together, as shown in Figure 1e and Figure S9 (Supporting Information). X‐ray tomography further confirms that the SEG possess a uniform, porous, and 3D structure, which is important for adsorption/desorption and transport of electrolyte ions (Figure S10, Supporting Information). By comparison, common laminar GO film also undergoes expansion into a porous structure under the same spark reaction but retains a long‐range ordered structure, characterized by horizontally arranged graphene sheets with large interlayer gaps (Figure 1f; Figure S11, Supporting Information). Moreover, the top‐view SEM image of laminar SEG reveals a continuous horizontal surface with few cracks (Figure 1h). By contrast, the surface of disordered SEG appears fragmented, featuring numerous intricate channels, conducive to facilitating the rapid ion infiltration into the electrode (Figure 1g).

Accordingly, the spark reaction imparts high porosity but low density to the disordered SEG. To solve this, a roll‐to‐roll compression process is developed to increase the density of the porous disordered SEG (Figure 1b–iii; Movie S2, Supporting Information). The flexibility of 2D graphene sheets and the highly porous structure endow the disordered SEG with excellent compressibility. As shown in Figure 1i,j, the porous structure can be significantly compressed from a thickness of more than 200 µm to less than 20 µm under ≈40 MPa compression pressure.

By comparison, despite the high compressibility of common porous graphene aerogels, they cannot achieve such dense structures solely through mechanical compression, mainly attributed to the strong bonding force between building blocks or the stiffening of graphene sheets in the traditional graphene aerogels during the gelation process.^[^ ^31^, ^32^ ^]^ Besides, the bulk density of compressed graphene films can be further controlled by adjusting the compression pressure.

**Figure 2:**
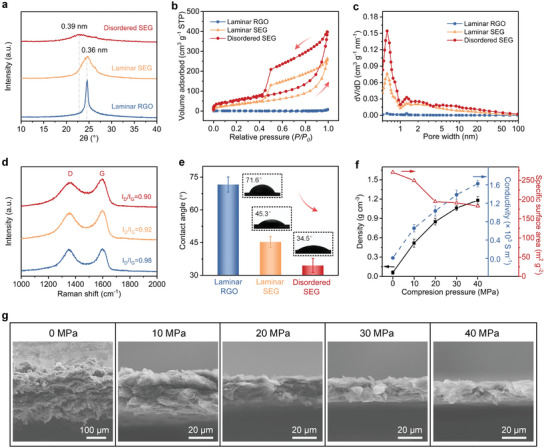
Characterization of disordered SEG film. a) XRD patterns of disordered SEG, laminar SEG, and laminar RGO films. (b and c) N_2_ adsorption‐desorption isotherms b) and corresponding pore‐size distribution c) of disordered SEG, laminar SEG, and laminar RGO films. d) Raman spectra of disordered SEG, laminar SEG, and laminar RGO films. e) Contact angles of an ionic liquid droplet (EMIMBF_4_, 5 µl) on laminar RGO, laminar SEG, and disordered SEG films. Of note, the disordered SEG and laminar SEG films have been subjected to 40 MPa compression pressure in (a–e). f) Density, specific surface area, and conductivity of the disordered SEG film at varying compression pressures from 0 to 40 MPa. The error bars denote the standard deviations of contact angle, density, and conductivity calculation from three independent samples. g) Cross‐sectional SEM images of disordered SEG films compressed under different pressures.

Compared to common electrode fabrication methods, the proposed expansion‐compression method in this work enables the simultaneous integration of fast ion diffusion capability, high ion‐accessible surface area, and high density in fabricated graphene films, conducive to achieving high‐performance compact capacitive energy storage. Moreover, this method offers several distinguishing advantages from a manufacturing perspective: (1) facile fabrication steps enabling the efficient fabrication of decimeter‐scale graphene electrode films (Figure 1k), (2) no reliance on monolayer graphene raw material, and (3) no toxic chemical reagents like hydrogen peroxide for chemical etching or hydrogen iodide for reduction and non‐active additives like binders or conductive agents. All these merits would be important for large‐scale manufacturing and practical capacitive energy storage applications.

### 2.2 Structure and Morphology Characterization of Disordered SEG

The internal structural features of the as‐prepared dense and disordered SEG film are examined by XRD. As shown in **Figure** **2**a, common laminar RGO film shows a sharp and intense characteristic peak at ≈24.5°, corresponding to a typical interlayer spacing of 0.36 nm. By comparison, the diffraction peak for laminar SEG film is obviously broader and weaker, indicating less stacking of graphene sheets within the film. For disordered SEG film, the diffraction peak further broadens, decreases in intensity, and slightly shifts to a lower angle of ≈22.8°, corresponding to an interlayer spacing of 0.39 nm. These differences in the XRD patterns suggest spark reaction enabling less stacking in graphene films. Moreover, the initial disordered arrangement of GO sheets enhances the spark reaction effect, as confirmed by the nitrogen adsorption‐desorption measurements. As shown in Figure 2b, laminar SEG film exhibits a significantly increased nitrogen adsorption quantity than common laminar RGO film, indicating less stacking of graphene sheets and the presence of more pores within the film. For disordered SEG film, the nitrogen adsorption quantity further increases, suggesting further increased porosity. The estimated Brunauer‐Emmett‐Teller (BET) surface of disordered SEG film is 183.2 m^2^ g^−1^, a value much higher than those of laminar RGO film (4.3 m^2^ g^−1^), and laminar SEG film (117.2 m^2^ g^−1^). In addition, both laminar SEG and disordered SEG films show a micropore‐dominated pore‐size distribution, where the predominant pores are ≈0.6 nm in width, dominating the amounts of EDL sites (Figure 2c).

Further details about the structures are obtained by Raman spectroscopy, as shown in Figure 2d. The Raman intensity ratios of the D to G bands (I_D_/I_G_) of disordered SEG, laminar SEG, and laminar RGO are 0.90, 0.92, and 0.98, respectively. The decrease in I_D_/I_G_ values of spark‐expanded films suggests that the exothermic spark reduction reaction slightly decreases the defect degree of graphene. In addition, the porous structures of graphene film originating from the spark reaction greatly improve its wettability to electrolytes, which is critical for an efficient penetration and spread of electrolyte ions.^[^ ^33^ ^]^ As shown in Figure 2e, the contact angle (CA) of an ionic liquid droplet on laminar RGO film reaches 71.6°, indicating the poor wettability of electrode film to the electrolyte. By comparison, laminar SEG film exhibits a smaller CA of 45.3° while disordered SEG film exhibits a further declined CA value to 34.5°, suggesting improved wettability.

By changing the compression pressure, the porosity and density of disordered SEG film can be further adjusted. Figure 2g compares the cross‐sectional SEM images of disordered SEG films subjected to various compression pressures from 0 to 40 MPa. As compression pressure increases, the arrangement of graphene sheets within the films becomes progressively more compact, accompanied by a gradual decrease in film thickness. Benefiting from the 3D interconnected porous structures and intrinsic flexibility of graphene, mechanical compression enables the elimination of large pores within the disordered SEG film without significantly modifying its microscopic porous characteristics.^[^ ^34^ ^]^ Nitrogen adsorption‐desorption measurements further reveal the variations in porosity for disordered SEG films under different compression pressures. As shown in Figure S12 (Supporting Information), the shrinkage of hysteresis loop in the isotherms indicates a gradual decrease in macropores and mesopores within the graphene films as the compression pressure increases from 0 to 40 MPa. However, the nitrogen adsorption quantity at relative pressure (*P/P_0_*) of less than 0.01 shows only a slight decrease, suggesting well‐preserved micropores during the compression process. As the compression pressure increases from 0 to 40 MPa, the BET surface decreases from 271 m^2^ g^−1^ to 183.2 m^2^ g^−1^ while the film density significantly improves from 0.06 to 1.18 g cm^−3^ and corresponding electrical conductivity increases from 11 to 1626 S m^−1^ (Figure 2f), which is conducive to high‐performance compact capacitive energy storage.

### 2.3 Electrochemical Properties of Disordered SEG

**Figure 3:**
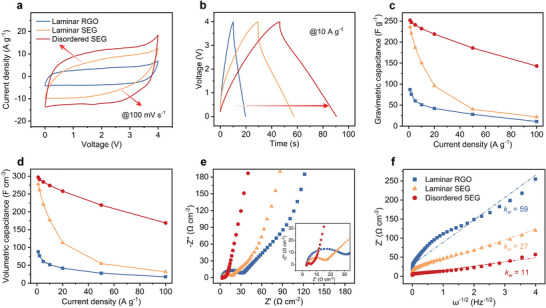
Electrochemical performance comparison of laminar RGO, laminar SEG, and disordered SEG films in EMIMBF_4_ electrolyte. a and b) CV curves at 100 mV s^−1^(a) and GCD curves at 10 A g^−1^ (b) of laminar RGO, laminar SEG and disordered SEG films. (c and d) Comparison of specific gravimetric (c) and specific volumetric (d) capacitances as a function of different current densities for laminar RGO, laminar SEG, and disordered SEG films. e and f) Nyquist plots (e) and Randle plots (f) of different graphene films over a frequency range from 100 kHz to 10 mHz. The inset in (e) is a magnified view of the high‐frequency region. Of note, disordered SEG and laminar SEG films have been subjected to 40 MPa compression pressure, and all graphene electrode films are tested using the same mass loading of 1 mg cm^−2^.

The electrochemical performances of disordered SEG, laminar RGO, and laminar SEG films are evaluated by assembling them into symmetric supercapacitors. In addition, a wide voltage window is the prerequisite for high energy and power densities, but the commonly used aqueous electrolytes (0 to 1 V) fall far short of meeting the high voltage requirement. For this reason, neat 1‐ethyl‐3‐methylimidazolium tetrafluoroborate (EMIMBF_4_) ionic liquid is selected as the electrolyte (0 to 4 V). The cyclic voltammetry (CV) curves at 100 mV s^−1^ and galvanostatic charge/discharge (GCD) curves at 10 A g^−1^ of the as‐obtained devices are compared in **Figure** **3**a,b, respectively. The nearly rectangular CV curves and triangular GCD curves indicate typical electrical double‐layer capacitive behaviors. Laminar RGO film exhibits the smallest CV curve‐enclosed areas and the shortest charge/discharge lasting times, indicating the poorest electrochemical performance. By comparison, the CV areas and charge/discharge lasting times obviously increase for laminar SEG film, and show further increases for disordered SEG film.

The specific gravimetric capacitances of these films derived from their GCD curves are provided in Figure 3c. Benefiting from the abundant micropores and disordered sheets orientation, the disordered SEG film shows higher ion accessibility and conductivity over laminar RGO and laminar SEG films, exhibiting the highest capacitance and rate ability. For example, disordered SEG film exhibits the highest gravimetric capacitance of 252 F g^−1^ at 1 A g^−1^ and retains 56.7% at a high current density of 100 A g^−1^. Laminar RGO film exhibits much lower gravimetric capacitance of 87 F g^−1^ at 1 A g^−1^ and retains only 18.4% at 100 A g^−1^. Laminar SEG film exhibits a high gravimetric capacitance of 236 F g^−1^ at 1 A g^−1^, but dramatically declines to 27 F g^−1^ at a current density of 100 A g^−1^, leaving only 11.4% of the initial performance. In addition, disordered SEG film also exhibits the highest volumetric capacitance of 297 F cm^−3^ at 1 A g^−1^, a value superior to those of laminar RGO (88 F cm^−3^ at 1 A g^−1^) and laminar SEG (278 F cm^−3^ at 1 A g^−1^) films (Figure 3d).

Further details about the rate capabilities of these films are provided by electrochemical impedance spectroscopy (EIS) analysis. The Nyquist plots over the frequency range from 100 kHz to 10 mHz are compared in Figure 3e. In the low‐frequency region, the plots feature quasi‐vertical curves, suggesting that all the different films show typical capacitive behaviors. In the high‐frequency region, the disordered SEG film shows a quite small semicircle and short 45° line (Figure 3e, inset), indicating fast ion transfer kinetics in the electrodes. By comparison, the length of the 45° line and diameter of the semicircle obviously increase for laminar SEG film, and both further increase for laminar RGO film, indicating an increased charge transfer resistance and decreased electrolyte diffusion efficiency.^[^ ^35^, ^36^ ^]^

The ion diffusion capabilities of the graphene films are quantified through the Randles plots, showing the dependence of impedance real part (Z’) on frequency (ω^−1/2^) (Figure 3f). The slope of Z’ versus ω^−1/2^ corresponds to the Warburg coefficient (*k_w_*), related to the ion diffusion coefficient (D) as follows:

(1) $$k_{w}=\frac{RT}{n^{2}F^{2}A\sqrt{2}}\left(\frac{1}{D^{1/2}C^{*}}\right)$$

where R represents the gas constant, T is the absolute temperature in Kelvin, n denotes the charge transfer number, A is the area of the electrode surface, and C* stands for the ionic concentration.

The slope of Randles plot decreases from 27 for laminar SEG film to 11 for disordered SEG film, suggesting a sixfold increase in ion diffusion coefficient for the disordered structure compared to common laminar structure. For laminar RGO film, the slop of Randles plot reaches as high as 59, suggesting quite sluggish electrolyte diffusion within the film.

### 2.4 Performance Optimization for Improved Compact Capacitive Energy Storage Applications

**Figure 4:**
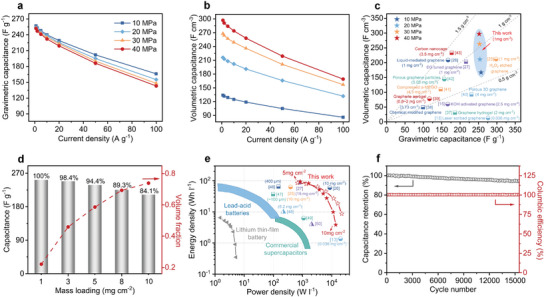
Optimization of the device performance for compact capacitive energy storage applications. a and b) Specific gravimetric (a) and specific volumetric (b) capacitances under different current densities for disordered SEG films prepared under different compression pressures. Of note, all graphene electrode films are tested with the same mass loading of 1 mg cm^−2^. c) Gravimetric and volumetric capacitance comparison of the disordered SEG films with reported electrode materials. d) Specific capacitance retention (bar chart) and volume fraction (dotted curve) of graphene electrode films with different areal mass loadings. e) Ragone plots of disordered SEG film‐based supercapacitors in comparison with previously reported values and state‐of‐the‐art energy storage devices (commercial supercapacitors, lead‐acid batteries, and lithium thin‐film batteries). All energy density and power density values are calculated based on the entire device configuration rather than only the electrode. f) Cycling stability of the supercapacitor devices over 15 000 cycles at a current density of 10 A g^−1^.

The above analyses demonstrated the superiority of disordered SEG film over laminar RGO and laminar SEG. Therefore, the capacitive performances of disordered SEG films prepared under different compression pressures (10, 20, 30, and 40 MPa) are studied for further optimization and application in compact capacitive energy storage. As shown in Figures S13 and S14 (Supporting Information), all disordered SEG films exhibit nearly rectangular CV and triangular GCD curves, indicating typical electrical double‐layer capacitive behaviors. **Figure** **4**a reveals that the disordered SEG film prepared under a relatively low compression pressure of 10 MPa achieves the highest capacitance value of 257 F g^−1^ at 1 A g^−1^, and the capacitance retains well as the compression pressure increases. For example, the disordered SEG film only exhibits a slight decrease in capacitance (252 F g^−1^ at 1 A g^−1^) as the compression pressure reaches 40 MPa, originating from the little restacking of micropores under compression as supported by the foregoing analysis on porosity. In addition, as the compression pressure increases from 10 to 40 MPa, the disordered SEG films show a certain decline in rate capabilities due to the decrease in mesopores and macropores within the graphene films. For example, the specific gravimetric capacitance of disordered SEG film prepared under 10 MPa compression pressure retains 64.6% (166 F g^−1^) of its initial performance when the current density reaches 100 A g^−1^, while only 56.7% (143 F g^−1^) is recorded for disordered SEG film prepared under 40 MPa compression pressure. However, when both the specific gravimetric capacitance and density are simultaneously taken into consideration, very different specific volumetric capacitances are recorded. For example, the increase in compression pressure from 10 to 40 MPa merely results in a modest decline of the specific gravimetric capacitance at a high current density of 100 A g^−1^ but yields a significant increase in density from 0.52 to 1.18 g cm^−3^. As a result, the disordered SEG film prepared under a high compression pressure of 40 MPa shows the highest volumetric capacitance of 169 F cm^−3^ even at the high current density of 100 A g^−1^, whereas disordered SEG film prepared under a low compression pressure of 10 MPa only yields a volumetric capacitance of 86 F cm^−3^ at 100 A g^−1^ (Figure 4b). Moreover, under 40 MPa, the disordered SEG film also yields the highest volumetric capacitance of 297 F cm^−3^ at a low current density of 1 A g^−1^. To the best of our knowledge, this specific volumetric capacitance is the highest among all reported carbon‐based electrodes,^[^ ^13^, ^15^, ^25^, ^26^, ^27^, ^37^, ^38^, ^39^, ^40^, ^41^, ^42^, ^43^ ^]^ exceeding the value of laser‐scribed graphene (13.2 F cm^−3^),^[^ ^13^ ^]^ KOH‐activated graphene (60 F cm^−3^),^[^ ^15^ ^]^ and H_2_O_2_‐etched graphene hydrogel (212 F cm^−3^)^[^ ^25^ ^]^ (Figure 4c).

Appropriately increasing the volumetric fraction of electrode material in the whole supercapacitor configuration is also essential to maximize the volumetric performance of the supercapacitors at the device level.^[^ ^44^ ^]^ Under the constant thickness of other components (e.g., current collector and separator) in supercapacitors, the volumetric fraction of electrode films can be increased by enhancing the electrode thickness, meaning the areal mass loading of electrode films. However, increasing the thickness of electrode films may degrade the capacitive performance due to more sluggish ion diffusion across the electrodes, especially for 2D materials with laminar structures.^[^ ^45^ ^]^ From this perspective, the disordered SEG film exhibits a high mass loading capability due to its highly porous structure and disordered sheet arrangement. For example, a high capacitance retention of 94.4% (238 F g^−1^ at 1 A g^−1^) is recorded for the disordered SEG film prepared under 40 MPa compression with a mass loading of 5 mg cm^−2^. As the mass loading increases to 10 mg cm^−2^ (common value reported for commercial carbon‐based supercapacitors), the electrode film still retains 84.1% of its capacity (212 F g^−1^ at 1 A g^−1^, Figure 4d). The increase in mass loading also improves the volumetric fraction of the electrode films in the whole supercapacitor configuration by more than threefold (from 0.22 to 0.74). Such a high volumetric fraction could be attributed to the absence of additives in the disordered SEG film, such as binders or conductive agents, preventing inactive components from occupying volume within the electrode films.

The excellent volumetric capacitance and high volumetric fraction contribute to the high volumetric performance of the supercapacitors at the device level fabricated using the disordered SEG films. As shown in Figure 4e, Figure S15 and Table S1 (Supporting Information), the fabricated stack cell with 10 mg cm^−2^ mass loading delivers an energy density of 94.2 Wh L^−1^ at a power density of 0.83 kW L^−1^, or 3.84 Wh L^−1^ at 13.7 kW L^−1^, which is tenfold higher than the energy density of commercial supercapacitors (5–8 Wh L^−1^) and exceeds the common energy density of acid‐lead batteries (50–90 Wh L^−1^), among the top level of state‐of‐the‐art carbon‐based symmetric supercapacitors.^[^ ^13^, ^15^, ^25^, ^26^, ^27^, ^46^, ^47^, ^48^, ^49^, ^50^ ^]^ For example, the energy density for a stack cell is 59.9 Wh L^−1^ at 8.6 kW L^−1^ for liquid‐mediated graphene,^[^ ^26^ ^]^ 63.2 Wh L^−1^ at 0.39 kW L^−1^ for H_2_O_2_‐etched graphene hydrogel^[^ ^25^ ^]^ and 88.1 Wh L^−1^ at 0.8 kW L^−1^ for EG‐tuned graphene.^[^ ^27^ ^]^ In addition, the stack cell with a lower mass loading of 5 mg cm^−2^ yields an areal capacitance of ≈ 0.6 F cm^−2^, meeting industrially acceptable areal capacitances.^[^ ^51^ ^]^ The stack cell with 5 mg cm^−2^ mass loading delivers a more balanced performance of 85.8 Wh L^−1^ at 0.67 kW L^−1^, or 8.7 Wh L^−1^ at 22 kW L^−1^ compared to the 10 mg cm^−2^ stack cell, enabling simultaneous capacitive energy storage with high‐energy and high‐power densities for wider applications. Moreover, the proposed disordered SEG‐based supercapacitor exhibits good charge‐discharge cycling stability with capacitance retention of 94.3% after 15 000 cycles at 10 A g^−1^ while maintaining a nearly 100% Coulombic efficiency (Figure 4f).

### 2.5 Fabrication of Flexible Solid‐State Supercapacitors

**Figure 5:**
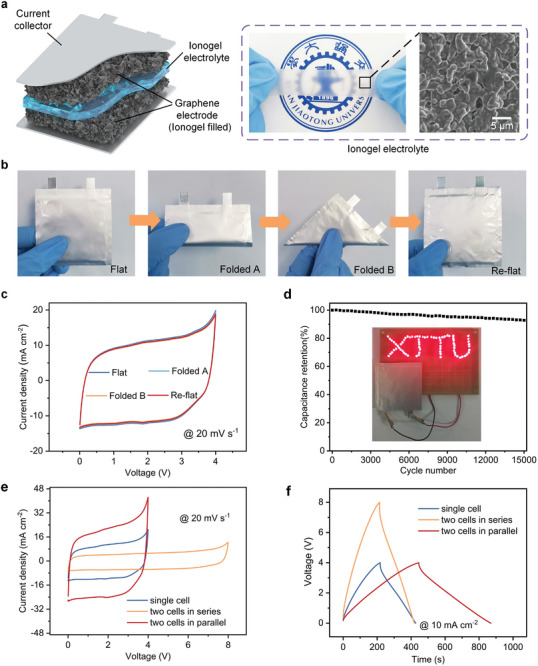
Fabrication of flexible solid‐state pouch cell using ionogel electrolyte. a) Schematic illustration of the pouch cell composed of the graphene electrodes, ionogel electrolyte, and aluminum current collector. The right side shows an optical photo and corresponding SEG image of solid‐state ionogel electrolyte. b and c) Photographs of a flexible pouch cell in flat, folded, and re‐flat states (b), along with the corresponding CV curves at a scan rate of 20 mV s^−1^ (c). d) Cycling stability of the pouch cell at a current density of 10 A g^−1^. The inset photograph shows an LED pattern composed of 74 diodes powered by the pouch cell. e and f) CV (e) and GCD (f) curves of the connection‐dependent outputs by two devices assembled in series and parallel.

To demonstrate potential applications in flexible electronic devices, flexible solid‐state supercapacitors are fabricated using disordered SEG graphene electrode films. **Figure** **5**a schematically illustrates the main construction of this pouch cell, where the solidified ionogel electrolyte, EMIMBF_4_/polyvinylidene fluoride‐hexafluoropropylene (PVDF‐HFP), is sandwiched between two graphene electrodes, acting as both the electrolyte and separator. Such a conformal connection endows the as‐assembled pouch cell with excellent robustness and flexibility, as demonstrated by Figure 5b. The CV curves exhibit nearly rectangular shapes and negligible changes when the pouch cell is repeatedly folded and re‐flatted (Figure 5c), further demonstrating excellent mechanical stability. In addition, the pouch cell shows great cycling stability maintaining 92.8% of its initial capacitance after 15 000 GCD cycles at a current density of 10 A g^−1^ (Figure 5d). As shown in Figure 5d (inset), a single pouch cell is capable of powering a light‐emitting diode (LED) pattern composed of 74 diodes assembled in parallel. Moreover, multiple optional outputs can be provided by configuring the pouch cells in different connections to meet different output requirements. As shown in Figure 5e,f, the series configuration extends the voltage window up to 8 V, while the parallel configuration provides twice the current of a single cell and delivers a high capacitance reaching ≈1 F cm^−2^. Altogether, the manufactured pouch cell from SEG disordered films suggests multiple merits in terms of wide operating voltage window, high mechanical stability, and optional outputs, demonstrating the enormous potential for use in flexible energy storage.

## 3 Conclusion

A novel method for fabricating disordered graphene with high ion accessibility, superior ion conductivity, and high density is successfully developed for high‐performance compact capacitive energy storage. Such porous and disordered graphene structure with a high ion‐accessible surface area and fast ion diffusion capability is readily fabricated by the spark‐induced expansion reaction of graphene oxide dough film. Subjection to further mechanical compression results in high‐density and disordered graphene while maintaining its porous characteristics due to the 3D interconnected structures and intrinsic flexibility of graphene material, making it an ideal electrode material for high‐performance compact capacitive energy storage. The resulting dense and disordered SEG film exhibits exceptional ion accessibility and fast ion diffusion with a high volumetric capacitance at the material level reaching 297 F cm^−3^ and a volumetric energy density at the device level reaching 94.2 Wh L^−1^, representing a milestone in capacitive energy storage. More importantly, the proposed method is facile, efficient, and scalable, thereby highly promising for large‐scale industrial manufacturing of capacitive energy storage devices.

## 4 Experimental Section

### Preparation of Disordered SEG Film

Industrial‐grade GO slurry (4 wt.%) was purchased from Shaanxi Coal Chemical Industry Technology Research Institute. The GO slurry was concentrated by stirring to facilitate water evaporation until reaching a dough‐like state (≈20 wt.%). A lump of GO “dough” was sandwiched between two diaphragms (Celgard 2500, 25 µm thick) and calendered into a film by a rolling machine (Shenzhen KEJING STAR Co., MSK‐HRP04B). The obtained GO dough film was then transferred into a plate press under a certain pressure until dried. The spark‐induced expansion and reduction reaction of the GO film was conducted by contacting the dough film with a 500 °C hot plate to yield a porous, disordered graphene “foam” (Movie S1, Supporting Information). Afterward, the obtained porous graphene “foam” was sandwiched between two aluminum foils, followed by roll‐to‐roll compression by the rolling machine (Movie S2, Supporting Information). The film density can be adjusted by controlling the compression pressure, meaning the distance of the gap between two rollers. For graphene film with a large areal mass loading (more than 5 mg cm^−2^), multiple graphene frameworks were stacked layer by layer and then calendered together.

### Preparation of Laminar SEG Film

GO dispersion (5 mg ml^−1^) was first prepared by diluting the GO slurry (4 wt.%). Next, deionized water (175 ml) was slowly added to the GO slurry (25 ml), followed by a gentle sonication for a few minutes. Laminar GO film was prepared by natural evaporation of the GO dispersion. Specifically, homogenous GO dispersion was poured into a glass petri dish and left at room temperature. As the water evaporated, the GO sheets were self‐assembled at the air‐liquid interface to ultimately form a free‐standing GO film. The resulting GO film was then peeled off from the glass petri dish and placed in a nitrogen‐filled glove box to conduct the spark‐induced expansion and reduction reaction, followed by a calendering process for densifying. The areal mass loading and density of the resultant film can be adjusted by changing the amount of GO dispersion per square centimeter and controlling the compression pressure, respectively.

### Preparation of Laminar RGO Film

Laminar RGO film was prepared by slowly annealing the as‐prepared laminar GO film. Specifically, the laminar GO film was sandwiched between two wafers and heated on a heating platform under a slow temperature rise rate of 5 °C min^−1^ until reaching a temperature of 500 °C.

### Preparation of Symmetrical Supercapacitor Cell

Commercially available carbon‐coated aluminum foil (Guangzhou Blueglownano, ≈15 µm thick) was first cleaned with ethanol for standby. The as‐prepared graphene film was then pressed onto the carbon‐coated aluminum foil under an appropriate pressure of 6 MPa to form graphene electrode film contacting the current collector, followed by drying at 120 °C for 2 h. Next, the graphene electrode film was punched into circular pieces with a diameter of 12 mm. Before the device assembly, the as‐prepared working electrodes were immersed into neat EMIMBBF_4_ ionic liquid under a vacuum for 12 h for full wetting. Afterward, two working electrodes with a separator (TF4030 NKK, 16 mm in diameter) were assembled in a CR2016 coin‐cell case. The whole assembly process of the supercapacitor cells was carried out in a nitrogen‐filled glove box with water content less than 0.01 ppm.

### Preparation of Flexible Solid‐State Pouch Cell

Flexible pouch cells were fabricated using aluminum laminate film (ALF) flexible package, graphene working electrodes, and EMIMBF_4_/PVDF‐HFP ionogel electrolyte. The preparation process consisted of several steps. 1) Preparation of the ionogel electrolyte by dissolving 1 g of PVDF‐HFP power (≈455 000 average Mw, Sigma–Aldrich) in acetone (20 ml) by magnetically stirring at 50 °C to yield a clear solution. EMIMBF_4_ (2 g) was then added to the above PVDF‐HFP solution and further stirred for 1 h at room temperature to form a homogeneous EMIMBF_4_/PVDF‐HFP ionogel electrolyte. 2) Filling the ionogel electrolyte into graphene electrode. To this end, the disordered graphene film with a mass loading of 5 mg cm^−2^ was first cut into 6 cm×6 cm square electrodes. The as‐prepared ionogel electrolyte solution was then cast onto the graphene electrodes and left to solidify at room temperature. Two pieces of graphene electrode were assembled together face‐to‐face using a drop of ionogel electrolyte as glue to form a working unit and packed in a pouch cell by a hot‐pressing process. All the above processes were carried out in a nitrogen‐filled glove box with a water content less than 0.01 ppm.

### Structural Characterization and Analysis

The morphologies of the graphene films were characterized by filed‐emission scanning electron microscopy (SU8010, HITACHI). X‐ray photoelectron spectroscopy was investigated with an AlKα source (Thermo Fisher ESCALAB Xi+). Fourier‐transform infrared spectroscopy measurements were carried out using a FTIR spectrometer (Thermo Fisher Nicolet iS10). X‐ray tomography was conducted with Xradia 610 Versa (ZEISS, Germany). X‐ray diffraction patterns were recorded on a Bruker D8 ADVANCE A25 instrument with CuKα radiation (λ = 1.54184 Å). Raman spectra were obtained on a HORIBA Laser Raman Spectrometer instrument with a laser wavelength of 532 nm. The N_2_ adsorption‐desorption isotherms were tested using Quantachrome Autosorb‐IQ at 77K. The specific surface area was calculated from isotherms based on the Brunauer‐Emmett‐Teller (BET) method. The pore size distribution was obtained by the Horvath‐Kawazoe (HK) method for micropores, and Barrett–Joyner–Halenda (BJH) method for mesopores and macropores. The conductivity of graphene electrodes was tested by the four‐probe method (Kaivo, FP‐001).

### Electrochemical Measurements and Calculations

The electrochemical measurements, including CV, GCD, and EIS, were conducted using a two‐electrode configuration on an electrochemical workstation (VersaSTAT 3, Princeton Applied Research, USA) at room temperature. The EIS tests were performed at open circuit potential under a sinusoidal signal over a frequency range from 100 kHz to 10 mHz with an amplitude of 10 mV. The cycle life tests were carried out by continuous GCD cycles at a current density of 10 A g^−1^.

The specific gravimetric capacitance (*C*_wt_) of a single electrode derived from GCD curves was calculated using Equation (2):

(2) $$C_{\mathrm{wt}}=\frac{2I\Delta t}{m\Delta U}$$

where *I* represents the constant discharge current, *Δt* is the discharge time, *m* stands for the mass loading of the electrode and *ΔU* is the voltage window (excluding the IR drop).

The corresponding specific volumetric capacitance (*C*_vol_) of a single electrode was calculated using Equation (3):

(3) $$C_{\mathrm{vol}}=C_{\mathrm{wt}}\times \rho$$

where *ρ* is the bulk density of the electrode active materials.

The gravimetric energy density (*E*_wt_) and volumetric energy density (*E*_vol_) of a single electrode were calculated by Equations (4) and (5), respectively:

(4) $$E_{\mathrm{wt}}=\frac{C_{\mathrm{wt}}\times U^{2}}{8}$$

(5) $$E_{\mathrm{vol}}=\frac{C_{\mathrm{vol}}\times U^{2}}{8}$$

where *U* is the operating voltage (excluding the IR drop).

The corresponding gravimetric power density (*P*_wt_) and volumetric power density (*P*_vol_) of electrode films were calculated by Equations (6) and (7), respectively:

(6) $$P_{\mathrm{wt}}=\frac{E_{\mathrm{wt}}}{\Delta t}$$

(7) $$P_{\mathrm{vol}}=\frac{E_{\mathrm{vol}}}{\Delta t}$$

where *Δt* represents the discharging time.

The gravimetric energy density (*E*_wt_) and volumetric energy density (*E*_vol_) of the entire device stack were obtained based on Equations (8) and (9), respectively:

(8) $$E_{\mathrm{wt}-\mathrm{stack}}=E_{\mathrm{wt}}\times f_{\mathrm{wt}-\mathrm{electrode}}$$

(9) $$E_{\mathrm{vol}-\mathrm{stack}}=E_{\mathrm{vol}}\times f_{\mathrm{vol}-\mathrm{electrode}}$$

where f_wt‐electrode_ and f_vol‐electrode_ are the mass fraction and volume fraction of the electrodes in the whole cell configuration (including both electrodes, separators, and current collectors). The experiments were carried out under the following conditions: ≈15 µm for each carbon‐coated aluminum foil current collector (≈4.2 mg cm^−2^), ≈30 µm for the NKK membrane separator (≈1.32 mg cm^−2^), and ≈85 µm for each graphene film with 10 mg cm^−2^ areal mass loading.

## Conflict of Interest

The authors declare no conflict of interest.

## Author Contributions

J.S., X.L., and G.L. conceived the idea and led research efforts. G.L. conducted the experiments. J.S. and X.L. supervised the project. G.L., X.L., C.L., Q.Z., Y.W., R.X., F.H., H.T., C.W., and X.C. helped with the experiments. G.L. drafted the manuscript. X.L. and J.S. polished the manuscript. All authors reviewed the manuscript.

## Supporting information

Supporting Information

Supplemental Movie 1

Supplemental Movie 2

## Data Availability

The data that support the findings of this study are available in the supplementary material of this article.
