# Supplementary material for: Efficient Fabrication of Disordered Graphene with Improved Ion Accessibility, Ion Conductivity, and Density for High‐Performance Compact Capacitive Energy Storage
Source: Adv Sci (Weinh). 2024 Aug 9;11(38):2405155. doi: 10.1002/advs.202405155 (PMC11481205; doi:10.1002/advs.202405155)
Supplement: Supplementary file 1 — Supporting Information [file ADVS-11-2405155-s002.pdf]

ADVANCED SCIENCE
Open Access

Supporting Information

for *Adv. Sci.*, DOI 10.1002/advs.202405155

Efficient Fabrication of Disordered Graphene with Improved Ion Accessibility, Ion Conductivity, and Density for High-Performance Compact Capacitive Energy Storage

Gangqiang Liu, Xiangming Li, Congming Li, Qinwen Zheng, Yingche Wang, Ronglin Xiao, Fei Huang, Hongmiao Tian, Chunhui Wang, Xiaoliang Chen and Jinyou Shao**

Supplementary Materials for

Efficient Fabrication of Disordered Graphene with Improved Ion Accessibility, Ion Conductivity, and Density for High-Performance Compact Capacitive Energy Storage

Gangqiang Liu, Xiangming Li, Congming Li, Qinwen Zheng, Yingche Wang, Ronglin Xiao, Fei Huang, Hongmiao Tian, Chunhui Wang, Xiaoliang Chen, Jinyou Shao**

Correspondence to (X. L.) xiangmingli@xjtu.edu.cn; (J. S.) jyshao@xjtu.edu.cn

This Supplementary Materials file includes:

Supplementary Figures S1 to S15

Supplementary Table S1

Supplementary References (1-11)

Other Supplementary Materials for this manuscript include the following:

Movie S1. Spark-induced expansion and reduction process of a GO film.

Movie S2. Roll-to-roll compression process for densifying a porous graphene film.

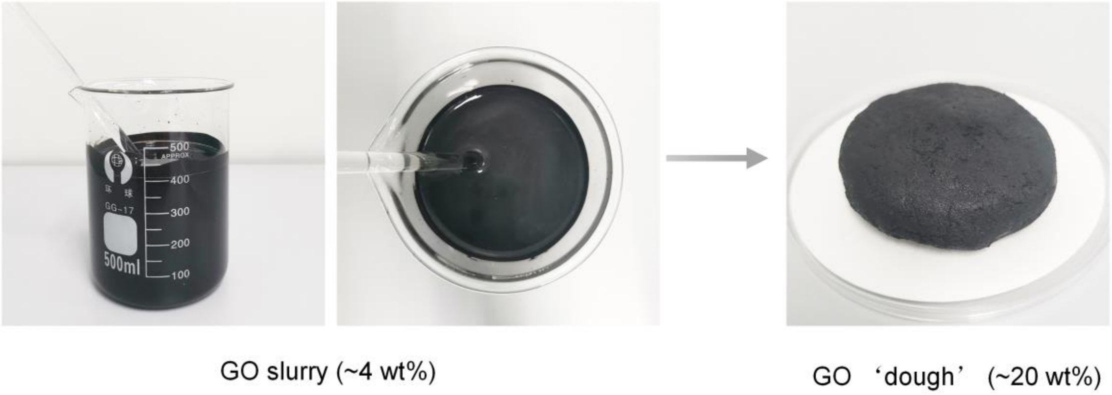

Figure S1. Photographs showing the transition of GO material from a flowable slurry (~4 wt%) to a malleable ‘dough’ (~20 wt%).

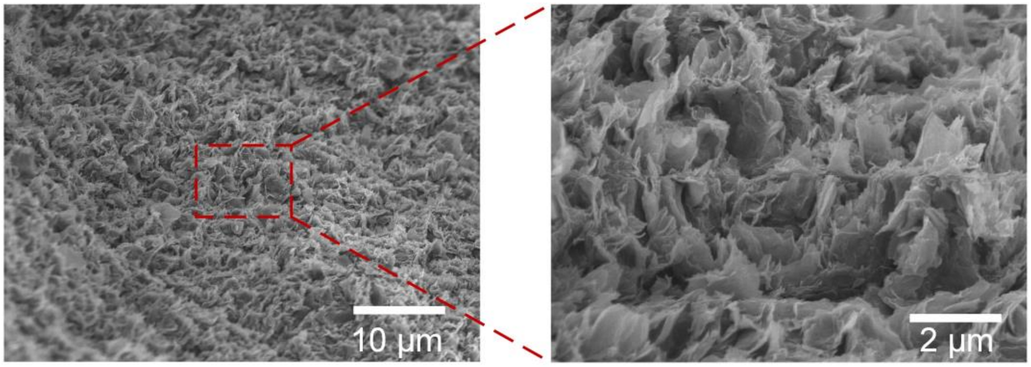

Figure S2. Cross-sectional SEM image of the dried GO ‘dough’.

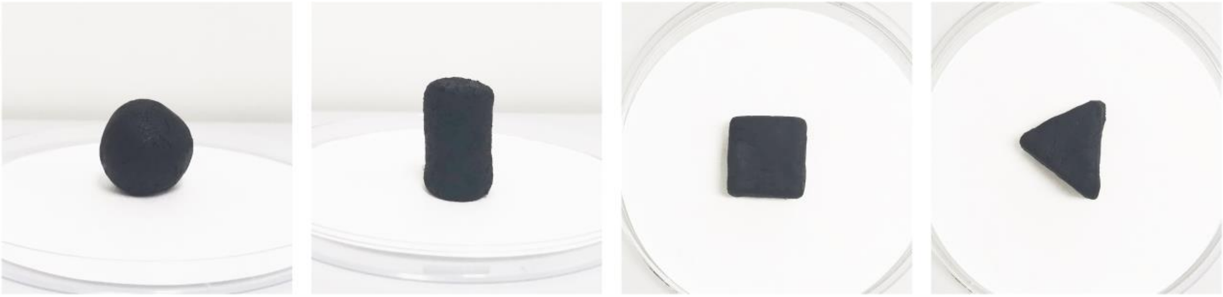

Figure S3. Photographs showing the processibility of GO ‘dough’ to form various shapes.

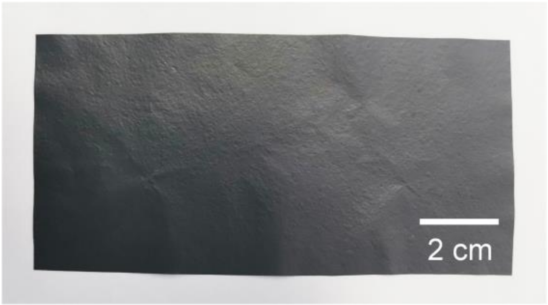

Figure S4. Photograph of a freestanding GO film prepared by calendering GO 'dough'.

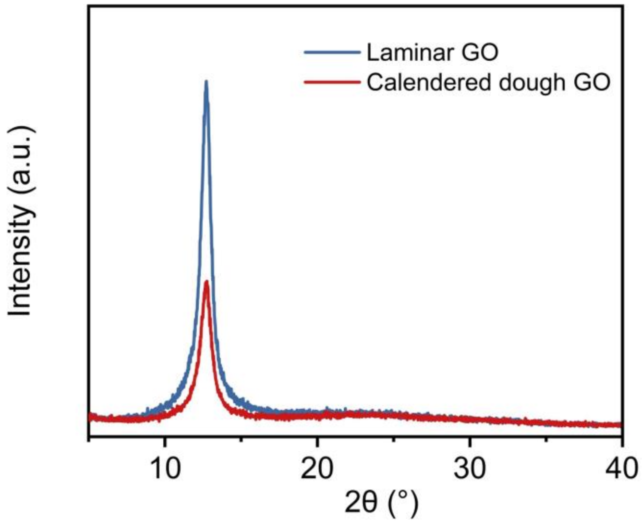

Figure S5. Comparison of XRD patterns between common laminar GO film and calendered GO dough film.

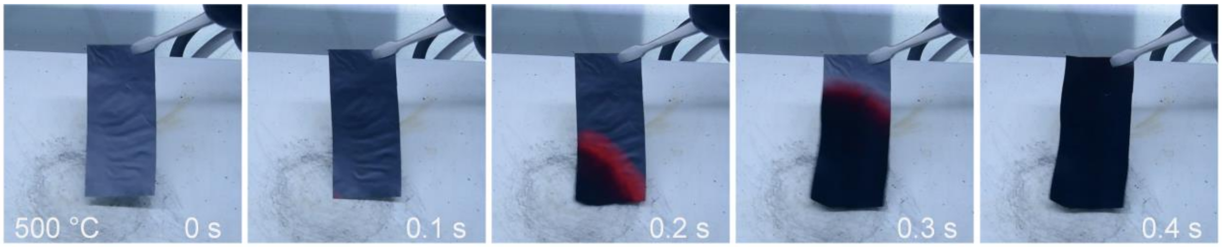

Figure S6. Photographs showing the spark-induced expansion and reduction process of a GO film.

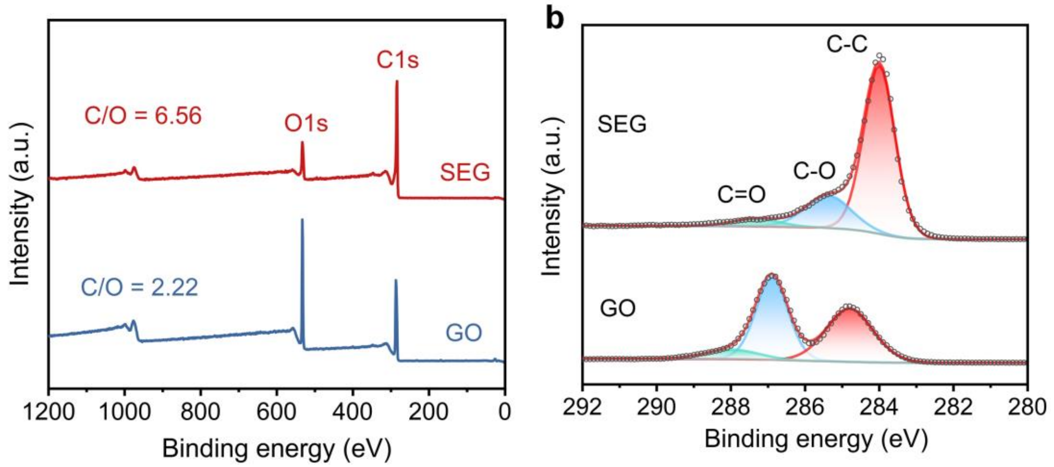

Figure S7. XPS characterization on GO and SEG. (a) XPS survey spectra and (b) C1s spectra. The C/O atomic ratios are 2.22, 6.56 for GO (blue) and SEG (red), respectively.

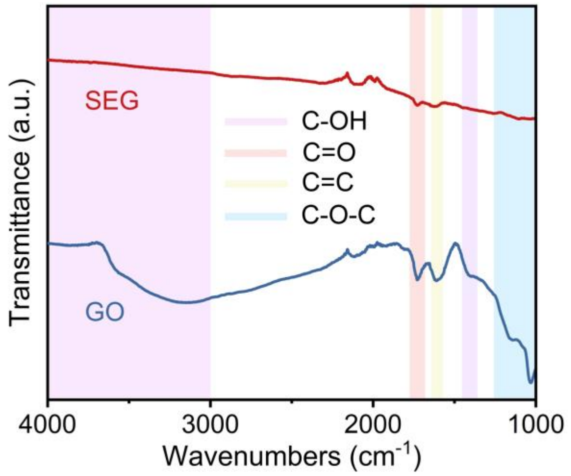

Figure S8. FT-IR spectra of GO (blue) and SEG (red).

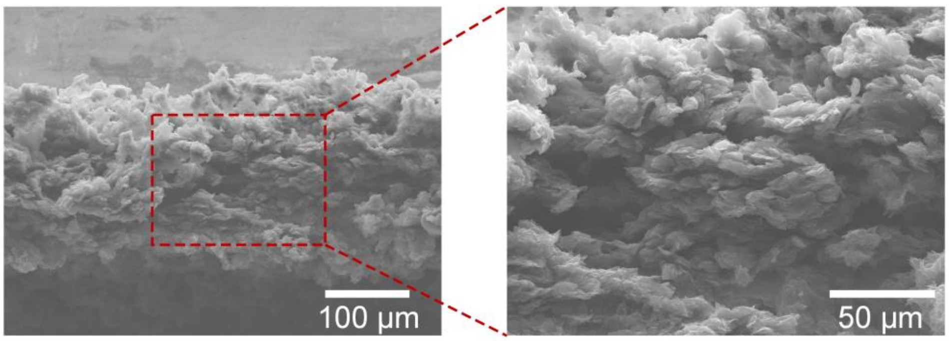

Figure S9. Cross-sectional SEM images of the expanded disordered SEG.

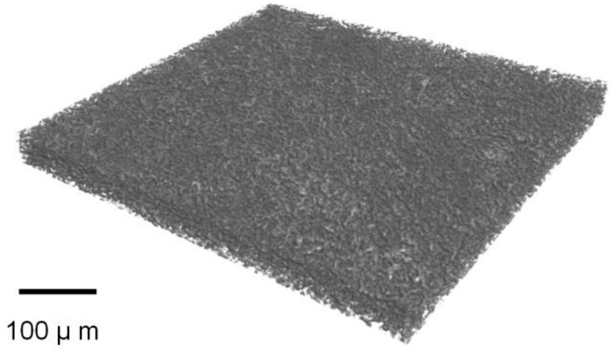

Figure S10. X-ray tomography of SEG. The result confirms that the SEG possess a uniform, porous, and three-dimensional structure.

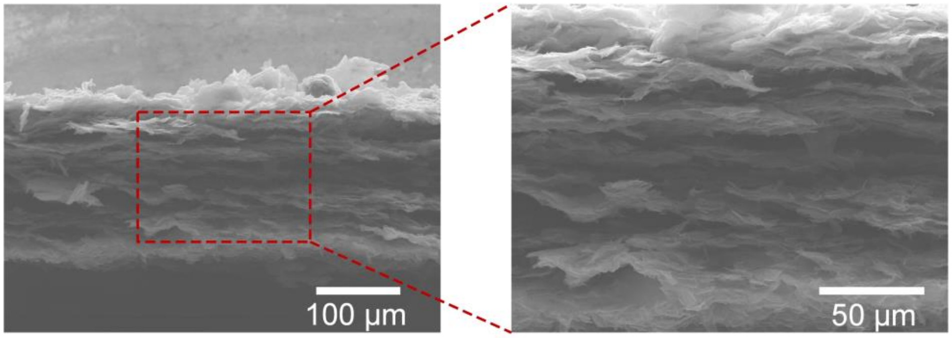

Figure S11. Cross-sectional SEM images of the expanded laminar SEG.

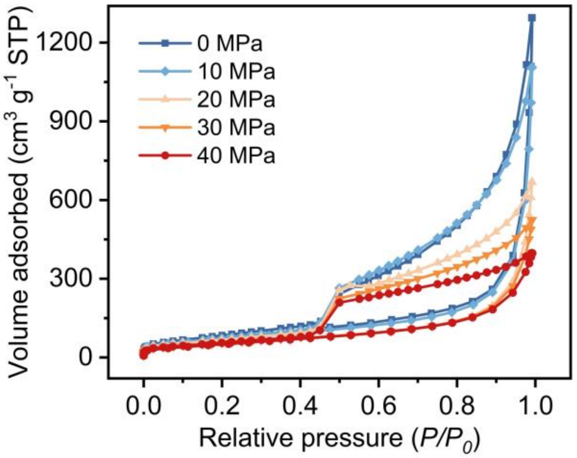

Figure S12. Adsorption-desorption isotherms of disordered SEG films under different compression pressures. The specific surface areas evaluated by Brunauer–Emmett–Teller (BET) method of these graphene films are 271 m^2 g^{-1} (0 MPa), 249.5 m^2 g^{-1} (10 MPa), 194.6 m^2 g^{-1} (20 MPa), 191.4 m^2 g^{-1} (30 MPa), 183.2 m^2 g^{-1} (40 MPa), respectively.

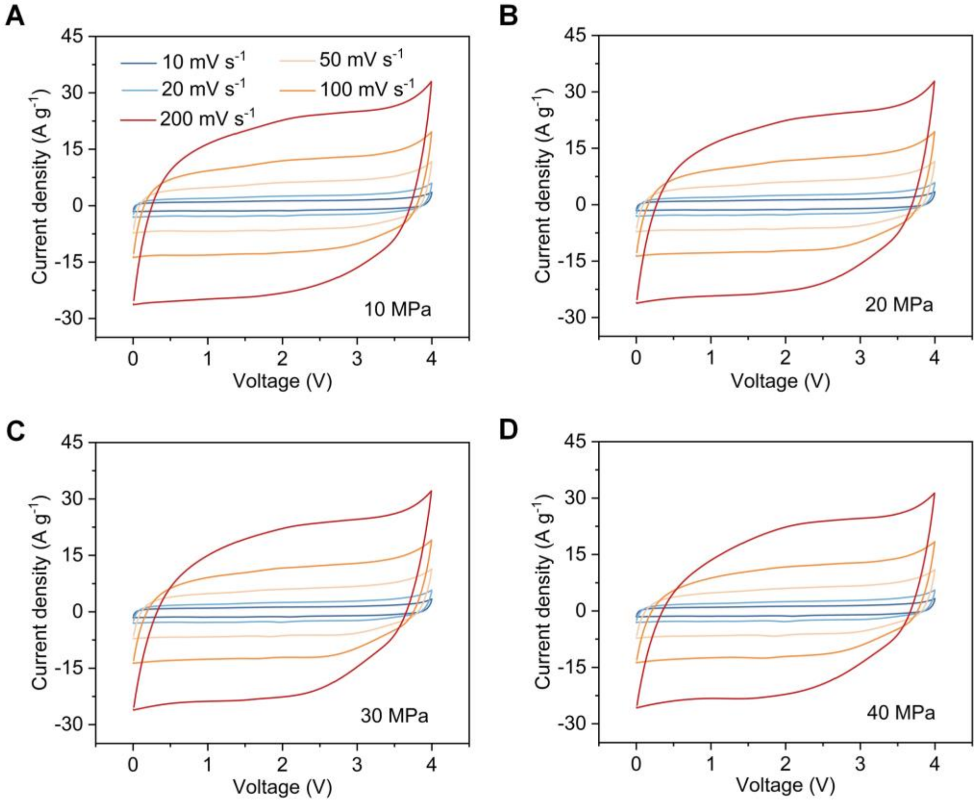

Figure S13. CV curves of disordered SEG films with different compression pressures. all graphene films were with the same mass loading of 1 mg cm^{-2}.

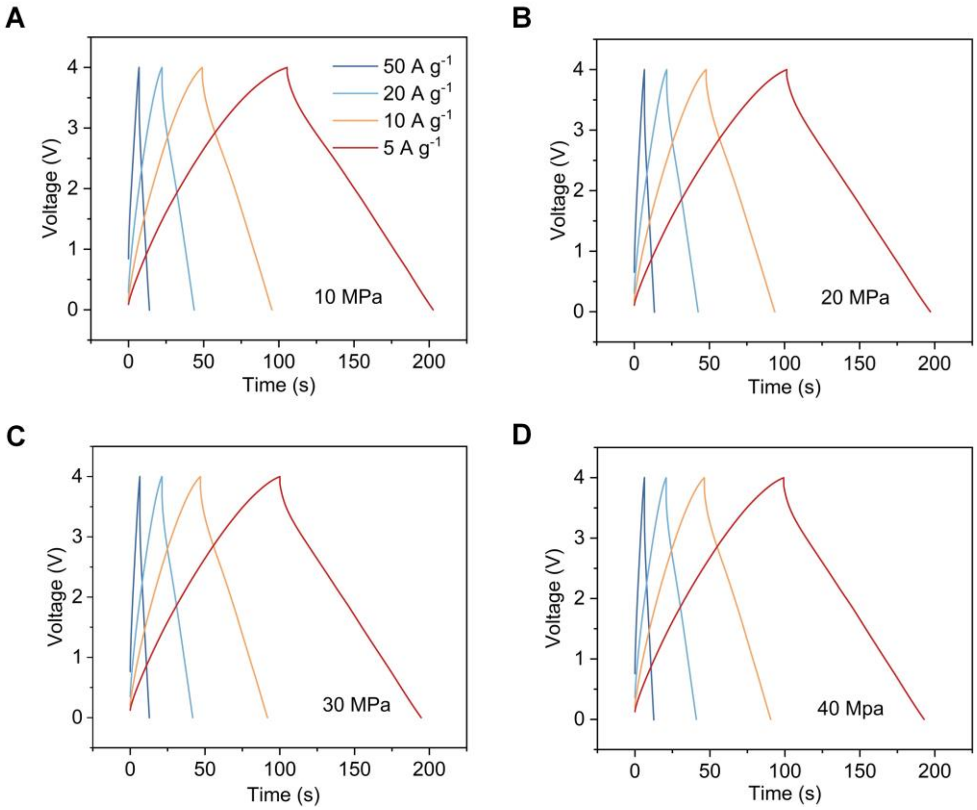

Figure S14. GCD curves of disordered SEG films with different compression pressures. all graphene films were with the same mass loading of 1 mg cm⁻

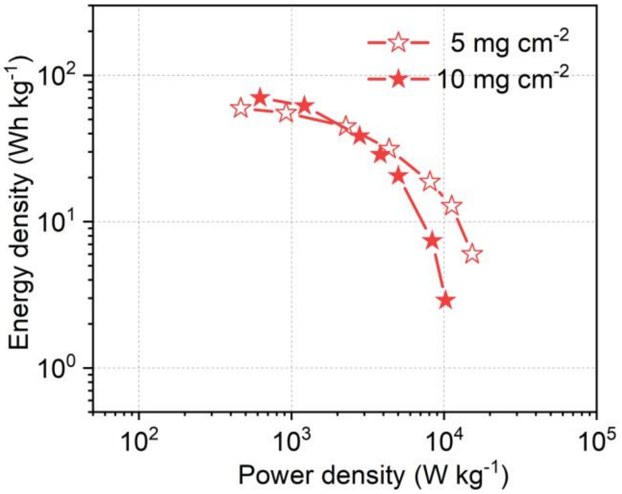

Figure S15. Ragone plot showing gravimetric energy density versus gravimetric power density of the stacked supercapacitors. For the device with electrode films of 10 mg cm^{-2}, the maximum energy density was as high as 70.2 Wh kg^{-1} at 622 W kg^{-1}, while a high-power density reached up to 10.2 kW kg^{-1} at an energy density of 2.9 Wh kg^{-1}. For the device with electrode films of 5 mg cm^{-2}, the maximum energy density was 59.6 Wh kg^{-1} at 465 W kg^{-1}, while a maximum power density reached up to 15.3 kW kg^{-1} at an energy density of 6 Wh kg^{-1}. Note that the weight fractions of the electrode films in the whole cell configuration were considered as ~0.65 and ~0.48 respectively.

Table S1. Performance comparison of various carbon-based supercapacitors.

Electrode materials	Mass Loading (mg cm^{-2})	Packing density (g cm^{-3})	Electrolyte (Voltage)	C_{wt} (F g^{-1})	C_{vol} (F cm^{-3})	$E_{wt\text{-}electrode}$ (Wh kg^{-1})	$E_{wt\text{-}electrode}$ (Wh L^{-1})	$E_{vol\text{-}stack}$ (Wh L^{-1})	Ref.
Disordered SEG	1	1.18	$EMIMBF_4$ (4.0 V)	252 (1.0 A g^{-1})	297	138	163	36	This work
	5			238 (1.0 A g^{-1})	281	124	146	85.8	
	10			212 (1.0 A g^{-1})	250	108	127	94.2	
Laser scribed graphene	0.036	0.048	$EMIMBF_4$ (3.5 V)	276 (5.0 A g^{-1})	13.2	117.4	5.6	1.06	[1]
KOH activated graphene	2.5	0.36	$BMIMBF_4$/AN (3.5 V)	166 (5.7 A g^{-1})	60	70	25	N/A	[2]
H_2O_2 etched graphene	1	0.71	$EMIMBF_4$/AN (3.5 V)	298 (1.0 A g^{-1})	212	127	90.1	25.7	[3]
	10			262 (1.0 A g^{-1})	186	112	79.5	63.2	
Liquid-mediated graphene	1	1.25	$EMIMBF_4$/AN (3.5 V)	167 (1.0 A g^{-1})	209	71	88.8	N/A	[4]
	10			126 (1.0 A g^{-1})	158	52	65	59.9	
EG tuned graphene	1	0.94	$EMIMBF_4$ (4.0 V)	216 (1.0 A g^{-1})	203	N/A	N/A	N/A	[5]
	15			201 (1.0 A g^{-1})	189	N/A	N/A	88.1	

Electrode materials	Mass Loading (mg cm^{-2})	Packing density (g cm^{-3})	Electrolyte (Voltage)	C_{wt} (F g^{-1})	C_{vol} (F cm^{-3})	$E_{wt\text{-}electrode}$ (Wh kg^{-1})	$E_{wt\text{-}electrode}$ (Wh L^{-1})	$E_{vol\text{-}stack}$ (Wh L^{-1})	Ref.
Compressed a-MEGO	4.3	0.75	$BMIMBF_4$/AN (3.5 V)	147 (1.2 A g^{-1})	110	63	47.3	N/A	[6]
Collapsed carbon nanocages	3.5	1.32	$EMIMBF_4$ (4.0 V)	177 (1.0 A g^{-1})	233	98	130	47.8	[7]
	10.6			156 (1.0 A g^{-1})	206	86	114	72.9	
Ultra-thick graphene	N/A	0.87	$BMIMBF_4$ (4.0 V)	172.4 (0.2 A g^{-1})	150	N/A	N/A	64.7	[8]
Graphene derived carbon	N/A	1.58	$TEABF_4$/AN (2.5 V)	106 (0.5 A g^{-1})	167	N/A	N/A	36.5	[9]
Highly aligned graphene	6.2	1.59	PVA/H_2SO_4 (1.0 V)	255	407	N/A	N/A	9.98	[10]
Carbon nanotube-graphene fibers	N/A	0.6	PVA/H_2PO_4 (1.0 V)	500	300	N/A	N/A	6.3	[11]

Abbreviations: SEG: spark-expanded graphene, EG: exfoliated graphene, a-MEGO: activated microwave-expanded graphite oxide. "N/A": Not available.

References

[1] M. F. El-Kady, V. Strong, S. Dubin, R. B. Kaner, *Science* **2012**, 335, 1326.

[2] Y. W. Zhu, S. Murali, M. D. Stoller, K. J. Ganesh, W. W. Cai, P. J. Ferreira, A. Pirkle, R. M. Wallace, K. A. Cychosz, M. Thommes, D. Su, E. A. Stach, R. S. Ruoff, *Science* **2011**, 332, 1537.

[3] Y. X. Xu, Z. Y. Liu, X. Zhong, X. Q. Huang, N. O. Weiss, Y. Huang, X. F. Duan, *Nat. Commun.* **2014**, 5, 4554.

[4] X. W. Yang, C. Cheng, Y. F. Wang, L. Qiu, D. Li, *Science* **2013**, 341, 534.

[5] Z. N. Li, S. Gadipelli, H. C. Li, C. A. Howard, D. J. L. Brett, P. R. Shearing, Z. X. Guo, I. P. Parkin, F. Li, *Nat. Energy* **2020**, 5, 160.

[6] S. Murali, N. Quarles, L. L. Zhang, J. R. Potts, Z. Q. Tan, Y. L. Lu, Y. W. Zhu, R. S. Ruoff, *Nano Energy* **2013**, 2, 764.

[7] Y. F. Bu, T. Sun, Y. J. Cai, L. Y. Du, O. Zhuo, L. J. Yang, Q. Wu, X. Z. Wang, Z. Hu, *Adv. Mater.* **2017**, 29, 1700470.

[8] H. Li, Y. Tao, X. Y. Zheng, J. Y. Luo, F. Y. Kang, H. M. Cheng, Q. H. Yang, *Energy Environ. Sci*. **2016**, 9, 3135.

[9] Y. Tao, X. Y. Xie, W. Lv, D. M. Tang, D. B. Kong, Z. H. Huang, H. Nishihara, T. Ishii, B. H. Li, D. Golberg, F. Y. Kang, T. Kyotani, Q. H. Yang, *Sci. Rep.* **2013**, 3, 2975.

[10] J. Zhong, W. Sun, Q. W. Wei, X. T. Qian, H. M. Cheng, W. C. Ren, *Nat. Commun.* **2018**, 9, 3484.

[11] D. S. Yu, K. Goh, H. Wang, L. Wei, W. C. Jiang, Q. Zhang, L. M. Dai, Y. Chen, *Nat. Nanotechnol.* **2014**, 9, 555.
